# Analysis of Liquid–Liquid Droplets Fission and Encapsulation in Single/Two Layer Microfluidic Devices Fabricated by Xurographic Method

**DOI:** 10.3390/mi8020049

**Published:** 2017-02-10

**Authors:** Chang Nong Lim, Kai Seng Koh, Yong Ren, Jit Kai Chin, Yong Shi, Yuying Yan

**Affiliations:** 1 Department of Chemical and Environmental Engineering, University of Nottingham Malaysia Campus, Jalan Broga, 43500 Semenyih, Selangor, Malaysia; kebx4lcn@nottingham.edu.my (C.N.L.); Jit-Kai.Chin@nottingham.edu.my (J.K.C.); 2 School of Engineering and Physical Sciences, Heriot-Watt University Malaysia, 62200 Putrajaya, Wilayah Persekutuan Putrajaya, Malaysia; 3 Department of Mechanical, Materials and Manufacturing Engineering, University of Nottingham Ningbo China, Ningbo 315100, China; yong.ren@nottingham.edu.cn (Y.R.); yong.shi@nottingham.edu.cn (Y.S.); 4 Research Centre for Fluids and Thermal Engineering, University of Nottingham Ningbo China, Ningbo 315100, China; yuying.yan@nottingham.ac.uk; 5 Research Group of Fluids and Thermal Engineering, Faculty of Engineering, University of Nottingham, Nottingham NG7 2RD, UK

**Keywords:** droplet manipulation, microfabrication, encapsulation, fission

## Abstract

This paper demonstrates a low cost fabrication approach for microscale droplet fission and encapsulation. Using a modified xurography method, rapid yet reliable microfluidic devices with flexible designs (single layer and double layer) are developed to enable spatial control of droplet manipulation. In this paper, two different designs are demonstrated, i.e., droplet fission (single layer) and droplet encapsulation (double layer). In addition, the current fabrication approach reduces the overall production interval with the introduction of a custom-made polydimethylsiloxane (PDMS) aligner. Apart from that, the fabricated device is able to generate daughter droplets with the coefficient of variance (CV) below 5% and double emulsions with CV maintained within 10% without involvement of complex surface wettability modification.

## 1. Introduction

Droplet microfluidics, one subcategory under microfluidics, enable the manipulation of fluid in the discrete form such as micro-droplets that offers greater benefits than the conventional flow systems in terms of high throughput and scalability. The generated droplets can be manipulated according to the desired droplet operations such as droplet fusion, fission, mixing and sorting [1,2,3,4]. On the other hand, compartmentalization of functionalized fluid within a droplet is useful for formation of double emulsions, microcapsules and microbubbles [5,6,7]. Droplet fission is known as a simpler way to introduce multiple sample arrays for rapid mixing or chemical reaction while increasing the throughput for droplet production and digitizing the biological assays at the same time [8], whereas controlled micro-encapsulation or better known as double emulsions provides a high entrapment efficiency and desired particle size [9]. On top of that, the core–shell structure enables the encapsulation and protection of a specific small volume captive ingredients for controlled released applications [10].

Over the past decade, a range of materials [11,12,13,14] has been reported to successfully fabricate the microfluidic device with different fabrication techniques developed. Such examples include soft-lithography [15], deep reactive-ion etching (DRIE) [16], micromachining [17] and rapid prototyping [18]. In addition, crucial factors in fabrication, such as process parameters and material bonding were also developed and optimized [19]. As such, xurography [20] technique was introduced as a new rapid prototyping method capable to reproduce soft microfluidic devices and glass microchannel [21] with higher reproducibility, easier installation and comparable lower overall fabrication cost than other techniques [22].

While polydimethylsiloxane (PDMS) is versatile, comparable low cost fabrication and allows easy replication with the presence of a master mold [23], the spatial wettability modification required is relatively difficult to achieve especially when it involves droplet encapsulation in a single piece device [24]. Microfluidic device made of glass capillary tube [14] has recently aroused as an established device for droplet encapsulation due to its advantages of conveniently functioned spatially and the precision in controlling the number and structure of encapsulation. However, the current fabrication procedure involves intricate operations, expensive equipment and mostly requires clean room facilities, thus inappropriate for mass production and commercialization [25]. Such a handicap has hindered the further stretch of droplet microfluidic development, hence motivates the aim of this paper which is to provide an alternative low cost fabrication method, xurography technique for droplet manipulation. In particular, this paper proposes a modified xerographic method by which a PDMS-Glass hybrid microfluidic device is fabricated enabling the process of droplet encapsulation to be carried out under consistent wall surface properties. This fabrication technique circumvents use of the complex surface wettability modification method, i.e., sol-gel coating method [26] and oxygen plasma [27], hence achieves environmental friendliness through the reduction of chemical usage, equipment and energy usage.

Specifically, we demonstrate in this paper the capability of xurography technique in fabricating single layer and double layer microfluidic devices with flexibility in channel dimension for different purposes of droplet manipulation, i.e., droplet fission (single layer) and droplet encapsulation (double layer). While the usual photolithography technique tends to produce a master mold with uneven channel heights due to the ultra-violet (UV) light curable duration, xurography technique discussed in this paper provides a constant channel height which is fixed by the thickness of the adhesive vinyl film used to replace silicon wafer in the photolithography technique. This provides distinguished advantages for hybrid devices such that a connection between different materials with fixed dimension is required. On top of that, the disposable adhesive vinyl film is cheaper than the single usage of silicon wafer, thus imposing higher economic efficiency in our xurography-based microfabrication portfolio.

The optimization for the fabrication of the single layer microfluidic device for droplet fission mainly involves the cutting plotter settings such as cutting force and offset. In contrast, the optimization for the sealing of the double layer droplet encapsulation device introduces the use of a custom-made PDMS aligner. The different settings of the cutting plotter and the detailed operation of the custom-made PDMS aligner are discussed to enable sharp edge corner cutting while ensuring the consistency of the alignment and sealing of the microfluidic device. The experimental results show different flowrate ratios to attest the functionality of the microfluidic device in which the droplets generated are all well maintained within 10% of the coefficient of variance (CV) value.

## 2. Materials and Methods

**Figure 1:**
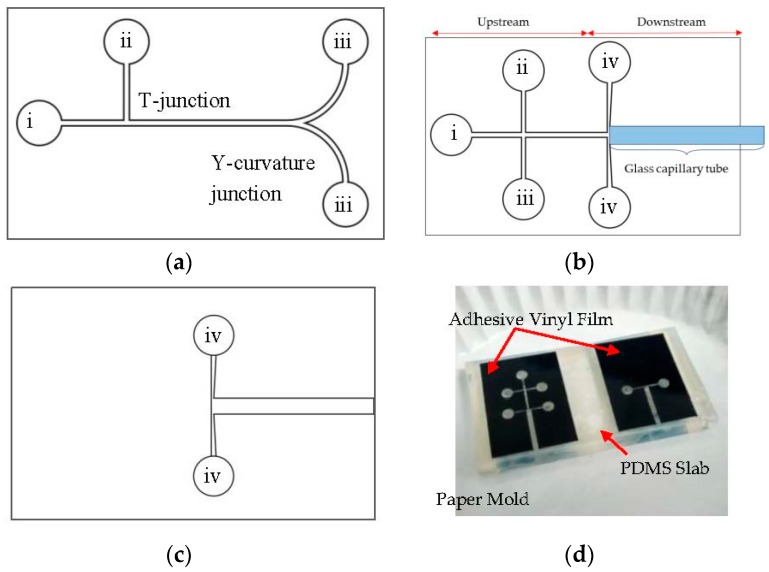
(**a**) Single layer microchannel design for droplet fission. The total length of the main channel is 28.10 mm with a 0.5 cm radii curve channel. (Label: i. cooking oil inlet; ii. DI water inlet; iii. Outlet). (**b**) Top layer design for droplet encapsulation. The channel height in the upstream is 0.2 mm. (Label: i. cooking oil inlet; ii. red dyed water inlet; iii. blue dyed water inlet; iv. yellow dyed water inlet). (**c**) Bottom layer design for droplet encapsulation to increase the downstream channel height to 1 mm for glass capillary tube fitting. (Label: iv. yellow dyed water inlet). (**d**) Layering for the fabrication of the epoxy master mold.

The modified xurography process started with a drawing of the microchannel design features (Figure 1a–c) using conventional drawing software. The design was then sent to the cutting plotter (CE6000-60, Graphtec, Yokohama, Japan) to be plotted onto 100 μm thickness adhesive vinyl film (Oracal Intermediate Cal 651, Orafol, Oranienburg, Germany). The vinyl film was then transferred to a blank PDMS slab which was later adhered onto a paper mold (Figure 1d). The epoxy resin and hardener (CP362 A/B, Oriental Option Sdn Bhd, Penang, Malaysia) were prepared at a ratio of 2:1 (*w*/*w*) and poured into the paper mold to be cured overnight at the room temperature (25 °C). PDMS pre-polymer (Sylgard 184, Dow Corning, Midland, MI, USA) prepared at a ratio of 10:1 (*w*/*w*) were poured into the master mold and left partial curing. The partially cured PDMS slabs were peeled from the master, aligned and sealed via a custom-made PDMS aligner. The double layer aligned PDMS layers was then heated for 2 h at 80 °C to complete.

For liquid–liquid droplet fission experiments, a T-junction geometry was utilized for water-in-oil (W/O) droplet generation. The W/O droplets were then directed towards the Y-curvature junction via the main channel to split into two daughter droplets (Figure 1a). The channel height was maintained at 0.1 mm throughout the whole device. On the contrary, droplet encapsulation device consists of two channel heights one is 0.2 mm at the upstream and the other is 1 mm at the downstream to enhance the encapsulation process. The dual T-junction were used at the upstream of the device to produce two distinct W/O inner droplets differentiated by color, i.e., red and blue which was then being encapsulated at the downstream forming a water-in-oil-in-water (W/O/W) double emulsions (Figure 1b,c). Glass capillary tube with an inner diameter of 0.58 mm (World Precision Instrument, Inc., Sarasota, FL, USA) was inserted at the downstream of the encapsulation device with the aid of the tweezer to create a hydrophilic environment for W/O/W double emulsions formation.

Droplet fission experiments were carried out with blended cooking oil as the continuous phase and pure deionized (DI) water (18.2 MΩ-cm, Milli-Q, Millipore, Molsheim, France) as the dispersed phase to generate W/O droplets. Droplet encapsulation experiments on the other hand, utilized cooking oil as the middle continuous phase while the two inner aqueous phase and outer aqueous phase were composed of a mixture of DI water with 16.6% (*v*/*v*), 9.0% (*v*/*v*) and 11.8% (*v*/*v*) of Fortune Red, True Blue and Egg Yellow food coloring, respectively, for W/O/W double emulsions. The materials used for the experimental work were pure cooking oil and DI water with food grade dye without addition of any other chemical solvent including surfactants. The materials were all used as purchased.

**Figure 2:**
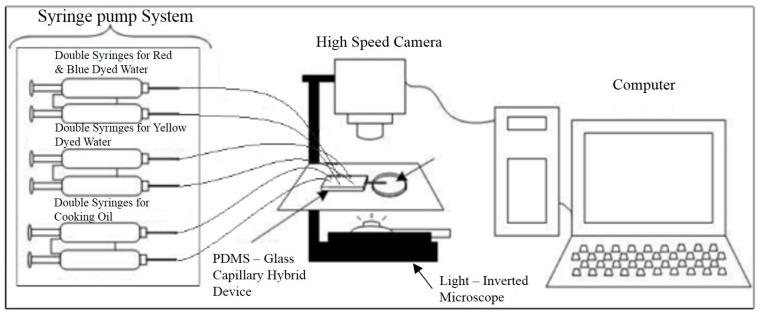
Schematic diagram of the experimental setup for droplet encapsulation and fission.

All the aforementioned liquids were loaded individually into their respective inlets through polytetrafluoroethylene (PTFE) tubing with an outer diameter of 1/16” (Omnifit^®^ Labware, Diba Industries Ltd., Cambridge, UK), delivered by syringe pumps (Model KDS-200, KD Scientific Inc., Holliston, MA, USA and NE-4000, New Era Pump Systems Inc., Farmingdale, NY, USA). The process of the droplet manipulation was recorded by a high-speed camera (Phantom Miro M110, Vision Research, Wayne, NJ, USA) mounted onto a light inverted microscope (Olympus IX51, Olympus Corporation, Tokyo, Japan) which was connected to a desktop (Figure 2). The droplet images were then extracted from the videos for a size distribution analysis via an image processing software—ImageJ (1.50i, National Institutes of Health, Bethesda, MD, USA). Experiments were repeated at least 3 times for the result accuracy purpose.

## 3. Results and Discussion

### 3.1. Fabrication Analysis

Previously, Pinto et al. [28] applied xurography to produce a biomedical microdevice. Since the xurography technique limits the minimum dimension of the microchannel width as 150 μm, they have compared the geometrical quality of the master mold with the corresponding microchannels using three sets of channel widths, i.e., 200, 300, and 500 μm. Their findings observe the largest inconsistence in channel width occurs at the smallest set of microchannels i.e., 200 μm with approximately of 50 μm difference. Therefore, in order to test on the increased accuracy and efficiency of the cutter plotter after optimization for xurography technique, the microchannel widths used in this study were mainly drawn in 200 μm. An orifice with a 150 μm channel width was also included for the optimization. 

#### 3.1.1. Cutter Plotter Optimization

Plotting quality can be directly affected by four main plotter settings: cutting force, offset value, offset angle and cutting mode. In this paper, cutter blade (CB09UB, Graphtec, Tokyo, Japan) was used to illustrate optimization of cutter setting on a 100 µm thickness vinyl film. New blade was used to carry out the optimization to prevent inconsistent results caused by blunted blade. The blade length was also adjusted and fixed to the optimum length using the built-in function. To tackle the problem systematically, the cutting force was first adjusted while keeping the offset and offset angle at default values.

**Figure 3:**
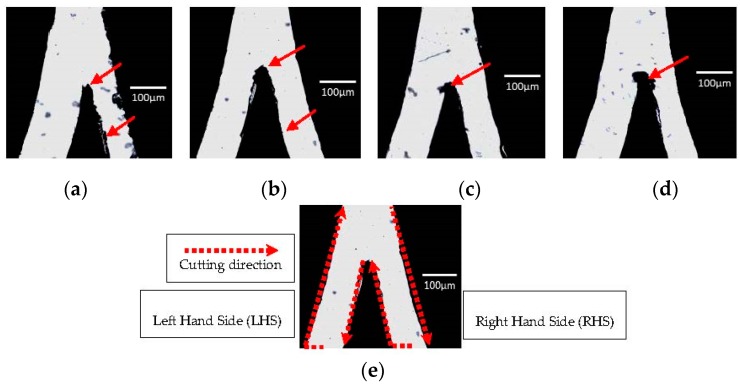
(**a**) Undercut vinyl film at the cutting force of 5; (**b**) vinyl film with the optimal cutting force of 12; (**c**) overcut vinyl film at the cutting force of 13; and (**d**) overcut vinyl film at the cutting force of 20, where the red arrows indicate the locations to be noticed on the sharpness of the cutting edge and the microchannel smoothness; and (**e**) the moving direction of the cutter blade when producing a microchannel.

The value of cutting force represents the pressure applied on the cutter blade to cut the design. Smooth cutting line can be obtained when the optimum cutting force is used. Starting with value 5, the setting was then gradually increased until traces of the blade were observed on the backing sheet. Figure 3 shows the appearance of the film at undercut, optimal and overcut condition, respectively. The width of the channel was measured with Image J and summarized in Table 1.

From visual inspection, the undercutting of the vinyl film was indicated by a rough channel surface with visible residue (Figure 3a). Since the pressure applied on the cutting blade was insufficient, the surface of the vinyl film was mostly dragged off by the blade resulting in a rough and torn pattern. On the contrary, excessive cutting force exerted onto the vinyl film affects the cutting area by indirectly ripping a larger shredded surface during the cutting process. At the optimal cutting force (Figure 3b), the channel surface presented is smooth and clean without visible residue as compared to the undercut and overcut vinyl films. The excessive cutting force applied has enlarged the inaccuracy caused by the offset and cutting mode, resulting in a blunt junction as indicated in Figure 3c,d. The moving direction of the blade when cutting this Y-curvature junction design further justifies the distortion towards the left (see Figure 3e). The measurement of the channel dimension is divided into two parts i.e., the left hand side (LHS) and right hand side (RHS) since a Y-curvature junction with 1:1 ratio was designed for this investigation purpose. The percentage error is calculated by |actual width − desired width|/(desired width) × 100%. We note that although the lowest percentage error is 0.2% at the value of 13 for the LHS, the cutting line is not as smooth (Figure 3c) as compared to the film cut at the value of 12 (Figure 3b). Hence, we conclude that the optimal cutting force is at the value of 12 with an error of 3.8% and 12.5% for LHS and RHS, respectively. Percentage error for the LHS falls under the range of 12%–38% due to the unequal width between both junctions. This can be resolved by further optimizing the offset or offset angle.

**Figure 4:**
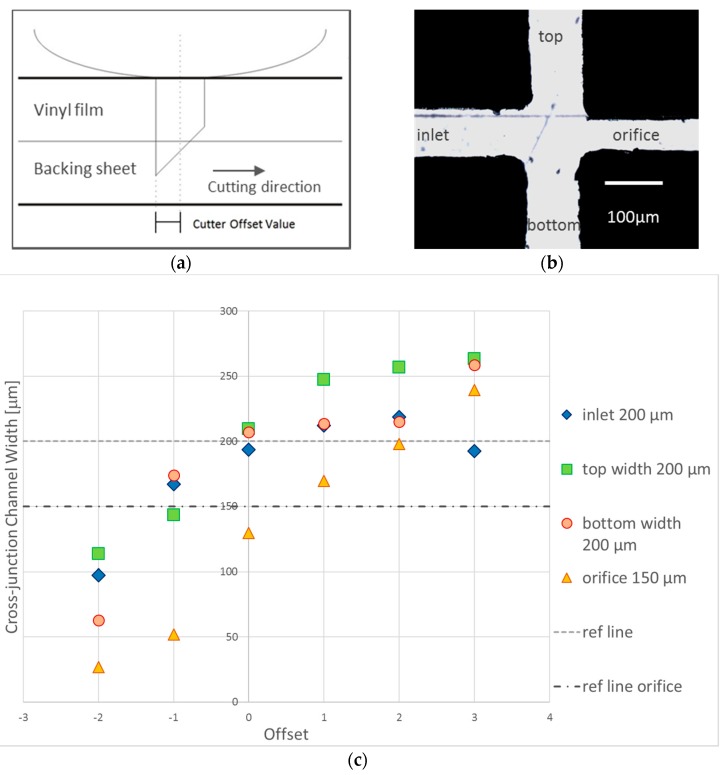
(**a**) Schematic illustration of the cutting mechanism of the cutter plotter; (**b**) vinyl film cutting at the optimal offset value 0; and (**c**) graph of cutting plotter offset versus cross-junction channel width. The reference (ref.) line refers to the desired width for the top, bottom and inlet channel i.e., 200 μm while the orifice width is at 150 μm. At the offset of 0 value, the errors of the channel widths produced towards the desired were below the 5% acceptable range.

The offset of a cutting plotter is defined as a distance difference between the center of the plunger and the tip of the blade in the plunger. The tip of the cutting blade is usually located after the center point due to the design of the blade (Figure 4a). Hence, fine-tune of the offset value allows the plotter to adjust the center point to an optimized coordinate for sharp cutting, especially at the bending of channel dimension. For better illustration, the Y-curvature junction was not used for offset adjustment. Instead, a cross-junction geometry of 200 μm width, with an orifice of 150 μm width, was used (Figure 4b). Since the cutting force had been optimized previously, the cutting force was kept constant at value 12 while the offset values in the range of −2 to +3 were tested with the offset angle and cutting mode set at default values. The channel width for each offset value tested was then measured and compared with its respective reference line (Figure 4c).

As the offset values were set towards the negative value, the centerline moved backwards to the tip of the blade. As a result, the blade turned after the desired cutting path causing a narrower channel with a closed orifice. On the other hand, while the offset value increased towards the positive value, the centerline moved further from the tip of the blade to its front resulting in the blade to turn before reaching the desired cutting path. This caused a wider channel and rounded corner. At the optimal offset value 0, the channel widths produced were close to the desired cutting dimension with acceptable percentage errors below 5%, i.e., 3.25%, 4.75% and 3.5% for the inlet, top and bottom channel respectively. These results show better consistency as compared to the experiments at the channel width of 200 μm reported in [28]. Although we notice an exception at the orifice in which the percentage error goes up to 13.7%, it is still the lowest as compared with the other offset values where the range of errors falls within 13%–65%. It is worth mentioning that sharp edged cutting was also well observed at the optimal offset value (Figure 4b).

**Figure 5:**
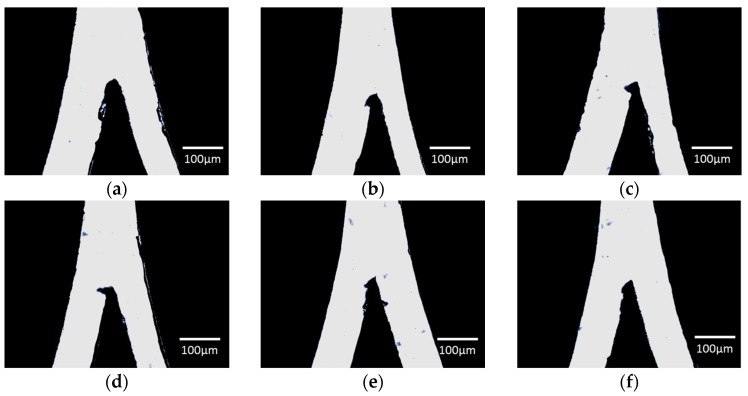
(**a**) Vinyl film cutting at the offset angle of 10°; (**b**) vinyl film cutting at the offset angle of 20°; (**c**) vinyl film cutting at the offset angle of 30°; (**d**) vinyl film cutting at the offset angle of 40°; (**e**) vinyl film cutting at the offset angle of 50°; and (**f**) vinyl film cutting at the offset angle of 60°.

The third factor we investigated is the offset angle, which is applied when there is a larger angle change than the specified reference angle. A larger value of the reference angle can reduce the plotting time by reducing the blade control time, however, insufficient angle control on the blade can occur if the value is higher than the optimum. The offset angles in our experiments were manipulated within a range of 10°–60° (Figure 5). When the offset angle increases, we observe that the width deviation between the two channels reduces until it reaches an equal channel width at both sides at the offset angle of 60°.

**Figure 6:**
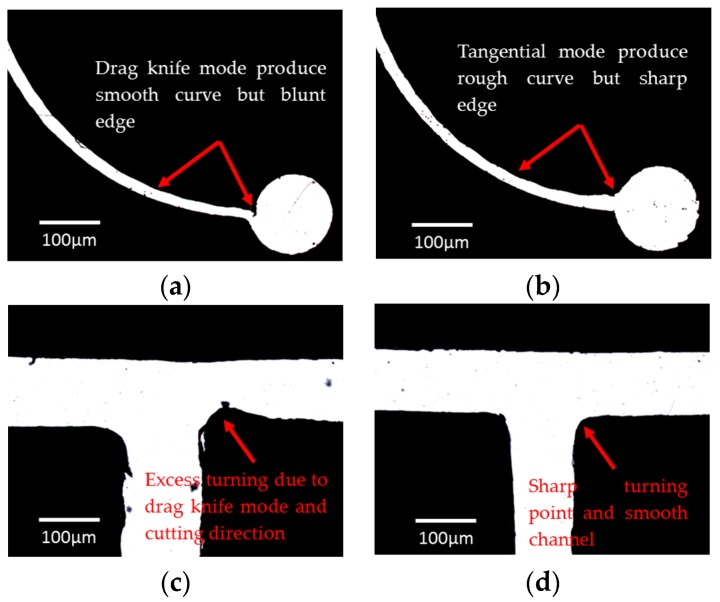
(**a**) Smooth channel curvature cut by the drag-knife mode. A clean cut near reservoir is harder to achieve in comparison to the tangential mode. (**b**) Rough channel curvature cut by the tangential mode with a cutting profile made of several straight lines with different angles. (**c**) Vinyl film with curvature at the corner by the drag-knife cutting even at the optimal offset value. (**d**) Clear edge cutting of the T-junction geometry by the tangential cutting.

Two cutting modes are available in the Graphtec cutting plotter CE6000-60, i.e., drag-knife and tangential mode. In the drag-knife mode, the blade cuts the vinyl film according to the path line, whereas, in the tangential mode, the blade is completely lifted from the film and rotates to a new position whenever the plotting involves a round curve and a sharp corner. The drag-knife mode (Figure 6a) is more appropriate when cutting a Y-curvature junction for a droplet fission device since the blade follows the cutting curve smoothly. This is different from the tangential mode where the blade lifts up whenever there is a bent. Therefore, the tangential mode can produce curves made up of lines with different angles, and provide a more precise and sharper edge cutting when straight channels are involved (Figure 6d). The drag-knife mode, on the other hand, gives a poor presentation when edge cutting is required (Figure 6c).

In short, the cutting mode depends on the shape of the channel design. The drag-knife mode is more appropriate when the design involves curves and arcs whereas the tangential mode is suitable when the design consists mostly of straight channels with sharp edge turning such as cross-junction and T-junction geometry. For a 100 μm thickness vinyl film, the optimal cutting force is at the value of 12 while the optimal offset value and offset angle are at the value of 0° and 60°, respectively. The optimization for both the cutting force and offset value was then extended to a double layer vinyl film where the optimal cutting force for the 200 μm thickness vinyl film was set equal to 16 while the optimal offset value was −2. However, a quantitative analysis is unable to be obtained in the current experiments as: (1) the cutting range of the cutter blade, CB09UB, is limited to a maximum of 250 μm; and (2) the thickness of available adhesive vinyl films is fixed at 100 μm.

Another technical limitation of the cutting plotter that affects the cutting quality is the cutting direction of the blade. Although the optimal conditions were achieved, small defects such as a slightly narrower channel width or a small area of excessive film at the turning edge, were still observed in our experiments. Since the cutting direction is fixed by the plotter, the only way to alter the direction is to rotate the orientation of the design before sending to the plotter. Consequently, the defects are hard to be eliminated thoroughly. However, shifting the design to another position can give the minimal impacts on the channel geometry.

#### 3.1.2. PDMS Aligner and Delta Analysis Results 

**Figure 7:**
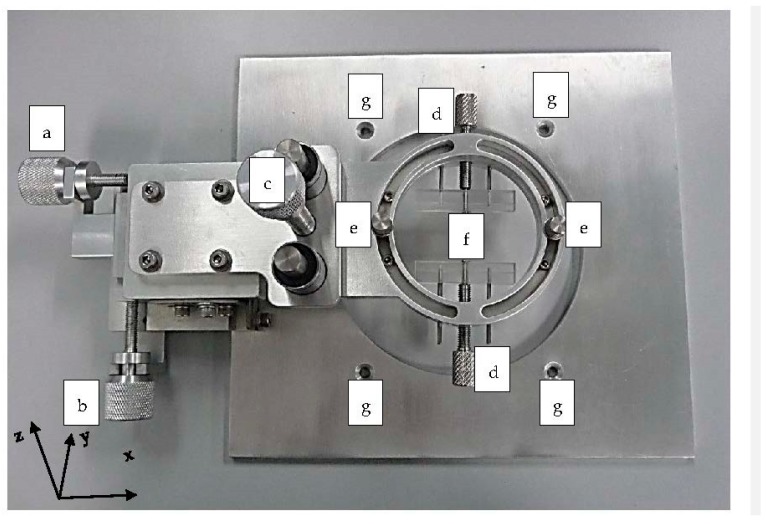
Image of the portable polydimethylsiloxane (PDMS) aligner with its functional knots that allow the PDMS layer to be moved in micro-meter (µm) intervals along the *x*, *y*, and *z* directions. (Labels: a. Left-right knot for PDMS slab’s left-right adjustment along the *x*-coordinates; b. Up-down knot to move PDMS slab’s up and down with micrometer intervals along the *y*-coordinates; c. Top-bottom knot to lower down the top layer of PDMS slab towards the bottom layer of PDMS slab along the *z*-coordinates and finally seal them together; d. PDMS-clip tightened knot for PDMS clip’s position adjustment; e. Skew adjustment knot to rotate PDMS slab in the *x*-*z* plane; f. PDMS clip to secure the position of the PDMS slab; and g. Screw hole to temporarily secure the device on the microscopic stage).

**Figure 8:**
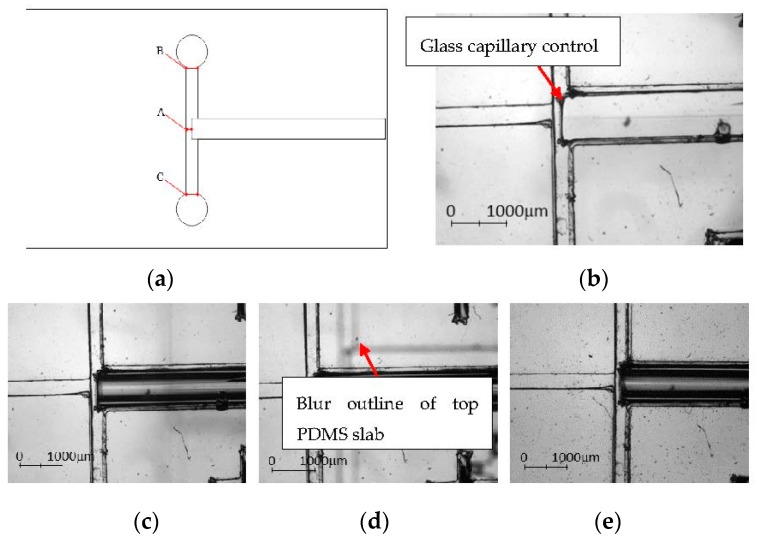
(**a**) Schematic diagram of the locations measured for the aligner precision analysis; (**b**) microscopic image of the bottom PDMS layer; (**c**) microscopic image of the bottom PDMS layer with glass capillary aligned on the channel; (**d**) microscopic image of aligning another layer of PDMS on the top; and (**e**) microscopic image of an aligned device.

The PDMS aligner (Figure 7) is made of stainless steel with 4 functional knots to adjust the PDMS layer in the *x*, *y*, *z* directions and allows rotation on the *x*-*z* plane by adjusting its skewness. In the alignment, the PDMS layer with a complex micro-channel design was placed onto the microscope stage as a base layer while the other PDMS layer was adjusted to the position by tuning the *x*-*z* knot until the edges of both layers were aligned accurately (Figure 8b–e). The entire process was completed under the light inverted microscope. 

The precision of the PDMS aligner was compared by measuring the channel width before and after the alignment process. The error and standard deviation were compared with bare-handed/manual alignment (Table 2). The channel widths of three locations (positions A, B and C in Figure 8a) were measured at the downstream of the encapsulation device, i.e., both the outer aqueous phase inlets and the distance between the glass capillary tube and the dispersion channel. To ensure repeatability and precision, aligner and manual alignment experiments were repeated by 10 times each and the average was calculated.

**Figure 9:**
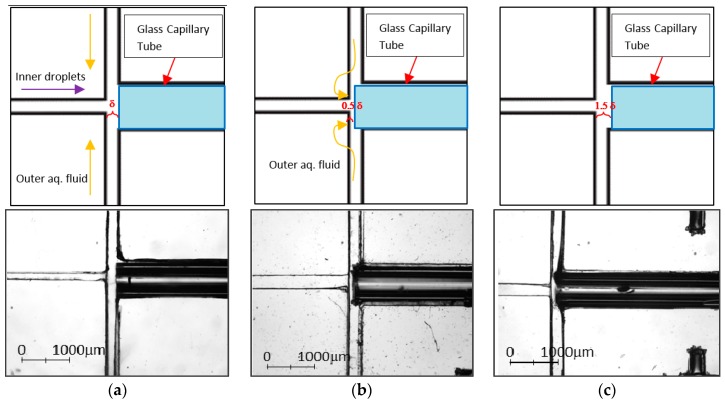
(**a**) Schematic illustration and the microscopic image of the glass capillary tube positioned at δ; (**b**) schematic illustration and the microscopic image of the glass capillary tube positioned at 0.5δ; and (**c**) schematic illustration and the microscopic image of the glass capillary tube positioned at 1.5δ. The glass capillary tube in the microscopic image dislocated due to expansion after heating. In the schematic diagram, yellow arrows indicate the flow of the outer aqueous phase according to different positions of the glass capillary tube while the purple arrow indicates the flow of the inner droplets.

In general, the error percentage of the alignment using the PDMS aligner is lower as compared to manual alignment except at Position A. However, this can be easily resolved by simply lifting the top PDMS piece without affecting the aligned coordinates to adjust the glass capillary tube back to its desired position with the aid of the tweezer. The position of the glass capillary is essential to ensure the success of the droplet encapsulation (Figure 9). For better demonstration, we use δ to indicate the channel width of the outer aqueous phase, i.e., the distance between the collection channel and the upstream dispersion channel. When the glass capillary tube was positioned at δ and 1.5δ, encapsulation was inconsistent as the pressure from the outer aqueous phase was insufficient to shear the incoming fluid. As the glass capillary tube moved towards the dispersion channel and was positioned at 0.5δ, the outer aqueous phase was forced to squeeze into the glass capillary tube. The pressure built up at the cross-junction allowed the formation of the encapsulated droplets.

### 3.2. Experimental Results—Droplet Encapsulation

The encapsulation experiments were conducted with four different flowrate ratios. The experimental data were grouped into two sets according to the manipulated flowrate ratios as shown in Table 3.

#### Relationship of Droplet Size with Flow Ratio, *Q*_disperse_/*Q*_continuous_

**Figure 10:**
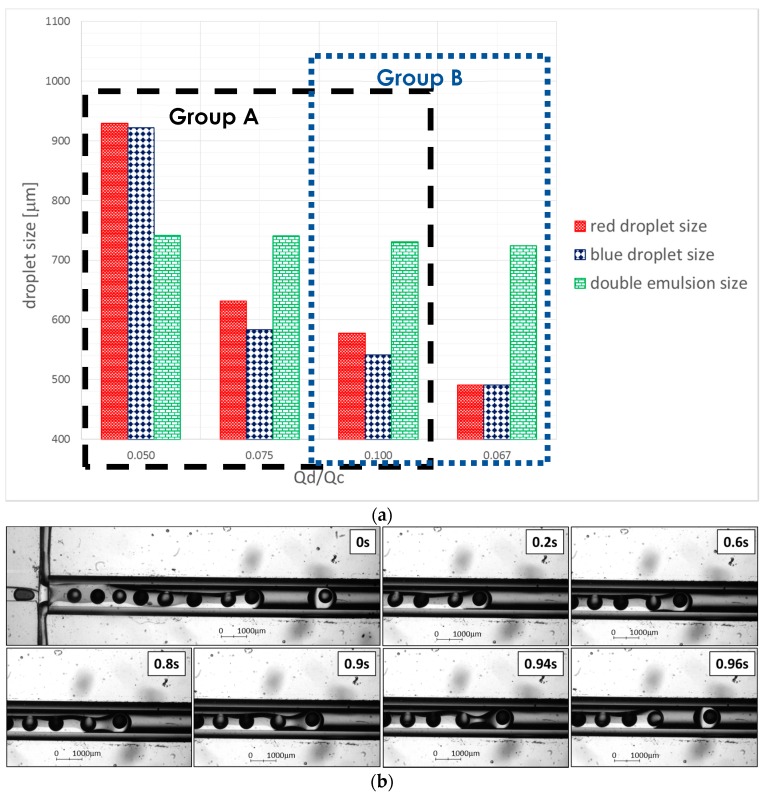

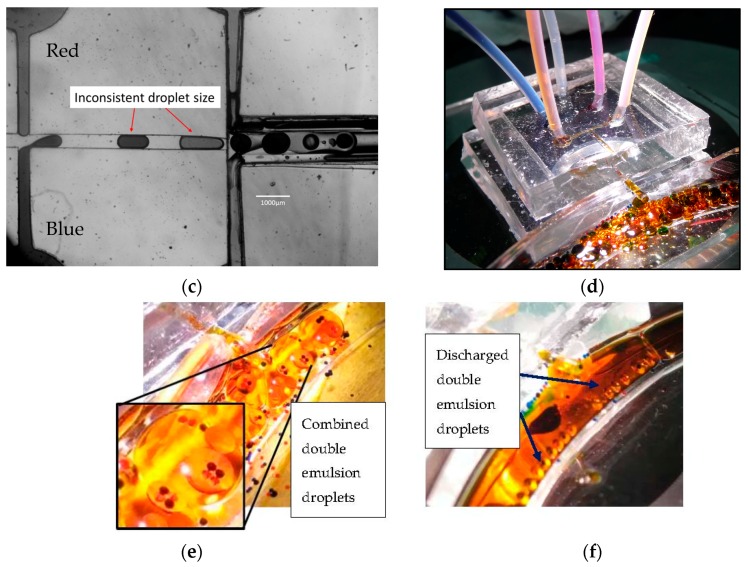
(**a**) Combination graph of droplet size versus flow ratio for Group A and Group B. Note that the values in the *y*-axis are not in an ascending trend since the sequence is in the ascending of flowrate ratio i.e., 1:2:40; 1:4:40; 1:6:40 and 1:6:60 µL/min. (**b**) Microscopic image of the droplet encapsulation process at the flowrate ratio of 1:6:40 µL/min. (**c**) Microscopic image of the droplet generation process at the flowrate ratio of 1:2:40 μL/min. (**d**) Image of the microfluidic device with the samples collected on the petri dish at the outlet. The double emulsion droplets will then slowly accumulate to form two layers of cooking oil and dyed water due to the absence of surfactant. (**e**) Image of the double emulsion droplets in the petri dish, two double emulsion droplets coalesced to form a larger droplet with four internal droplets due to the continuous flow disturbance from the outlet. (**f**) Image of the double emulsion droplets after discharged from the device with mostly single internal droplet encapsulated.

The flow regimes for the droplet generation are mainly distinguished by the capillary number of the continuous phase, Ca_c_. Whilst Ca_c_ < 0.002 indicates the squeezing regime, 0.002 < Ca_c_ < 0.01 indicates the transient regime and 0.01 < Ca_c_ < 0.3 indicates the dripping regime [29]. The Ca_c_ at all flowrates were maintained at 0.0001 in our experiments indicating the squeezing regime for the downstream encapsulation process, while the droplet generation process at the upstream falls into the transient flow regime. Therefore, the droplet size depends predominantly on the flow ratio, *Q*_disperse_/*Q*_continuous_, instead of Ca_c_ [30]. Hence, Figure 10a shows the relationship between the droplet size and the flow ratio. Figure 10b illustrates the animation of droplet generation and encapsulation process of forming double emulsion droplets while Figure 10c shows the inconsistent droplet sizes formed due to the disproportion dominant force acting on droplet break up. The double emulsion droplets formed in the hybrid device were all discharged onto the petri dish as shown in Figure 10d–f.

Since the droplet generation at flowrate ratio 1:2:40 µL/min falls under the transient regime, the dynamics of droplets break up would be dominated by both the shear force and interfacial force [29]. However, as Ca_c_ in these cases are very close to the squeezing regime, i.e., 0.003, the disproportion in both dominant forces i.e., shearing from the continuous phase and pressure built up across the inner droplets, causes the inconsistency of the internal droplet sizes (Figure 10c) with high values of CV i.e., 17.3% and 20.1% for red and blue droplets, respectively. From the experimental observations, a long water plug was formed when interfacial force dominated over the shear force and vice versa. Hence, this results in the formation of polydispersed internal droplets at the upstream of the device.

On the other hand, both inner droplet sizes show inconsistency at 1:4:40 (CV = 8% and 9.7%) and 1:6:40 µL/min (CV = 9.9% and 13.9%), even though the channel dimensions and inner flowrates were kept constant in these two cases. The experimental observations show a competition between both inner droplets squeezing out from their respective channel. This leads to two conditions: (1) the tip of the inner droplet was forced reverse to its channel by the opposite inner fluid before it could further elongate to the main channel and break off; and (2) the inner droplet had been sheared by the opposite inner fluid before it was fully developed to break up as a droplet. The first condition disrupts and randomizes the alternate sequencing of the droplets. The second condition, however, as the main reason influencing the consistency of the droplet production, results in a higher coefficient of variance (CV). The deviation between the two inner droplet sizes was calculated by |blue droplet diameter − red droplet diameter|⁄(red droplet diameter) × 100%. The results obtained are 0.8%, 7.5% and 6.4% respective to *Q*_disperse_/*Q*_continuous_ of 0.050, 0.075 and 0.100 (Figure 10a). 

In group B, the inner droplet size of the 1:6:60 µL/min decreased significantly with higher similarity in size (CV = 5.9% and 9.1%). The competition between the droplets at the upstream was no longer observed once the system was stabilized. Only 0.1% difference between the red and blue inner droplet sizes was seen. This further evidences the influences and interactions among the interfacial forces of the inner, middle and outer fluids in the droplet encapsulation system. Current approach has produced monodispersed double emulsion with CV maintained at approximately or less than 10%. The double emulsion size remained at a range of 723–741 µm. While 1:6:60 µL/min possessed the smallest double emulsion size, flowrate ratio of 1:2:40 µL/min has the largest emulsion size. The CV percentages of all flowrate ratios were tabulated in Table 4. 

With different flowrate ratios, six different types of encapsulation are observed from the experimental work: (i) two distinct droplets with one red and one blue droplet encapsulated; (ii) two similar droplets with either two red; or (iii) two blue droplets encapsulated; (iv) single droplet encapsulation with either one red; or (v) one blue; and (vi) zero encapsulation which occurs whenever there is a flow disturbance from the syringe pumps. The encapsulation percentage/success rate of the double emulsion droplets formation are tabulated in Table 5.

Ultimately, distinct droplet encapsulation is preferred for better control and application wise. However, from an overall comparison, most of the flowrate ratios favors the single droplet encapsulation except for 1:6:60 µL/min which shows a higher total percentage in the double droplet encapsulation. Comparing different cases in group A, 1:4:40 µL/min shows the highest single encapsulation among the three flowrate ratios while 1:2:40 µL/min has the highest total percentage in double droplet encapsulation. At 1:2:40 µL/min, the internal droplets generated are in a longer plug size, i.e., 920 μm, with smaller spacing between the droplets. This narrow spacing between the droplets and low droplet generation frequency lead to a higher percentage in encapsulating two internal droplets. When the middle phase flowrate increased to 4 µL/min, the spacing between the droplets generated increased. This is because the middle continuous phase increases the flowing velocity of the generated droplet. However, the shear force was insufficient to increase the frequency of droplet production simultaneously, and thus leading to a higher percentage of single droplet encapsulation. As the middle phase flowrate further increased to 6 µL/min, the droplet spacing reduced with increasing droplet frequency. Therefore, we obtained a higher percentage of double droplets encapsulation at 1:6:40 µL/min.

The outer aqueous flowrate was then further increased to 60 µL/min with a constant inner and middle flowrates. Result shows the total percentage of double droplet encapsulation in this case is the highest at 61.5% with the lowest single droplet encapsulation at 38.5%. Until current stage of experiments, flowrate ratio 1:6:60 µL/min would be the optimum for encapsulating two distinct droplets. On top of that, no coalescence was observed between the two inner droplets though no surfactants were used. We attribute to the larger channel dimension of the glass capillary tube, where the droplet dimension is no longer constraint by the microchannel width and hence no coalescence occurs.

### 3.3. Experimental Results—Droplet Fission

**Figure 11:**
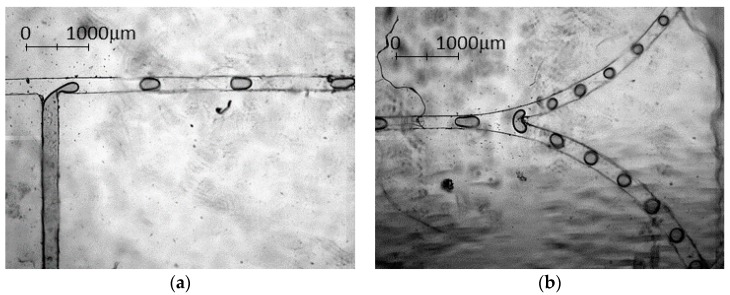
Droplet fission from the high speed camera of 1:1 curvature ratio at dispersed flowrate of 2 µL/min and continuous flowrate of 6 µL/min: (**a**) water droplet breakup at the T-junction; and (**b**) the droplet fission where a mother droplet breaks up into two daughter droplets. The blurriness of the images was caused by the cooking oil stain, which adhered to the top of the device surface during the experiment.

Next, the droplet fission experiments will be further discussed. The droplet fission phenomenon started with a conventional upstream T-junction droplet formation [30] as shown in Figure 11a. Upon reaching the Y-curvature junction downstream, the mother droplet (Zone A, Figure 11b) experiences a competition among various forces subject to microchannel and liquid properties. The surface tension, originated from the dispersed water phase underwent stretching caused by uneven microchannel pressure distribution [30] and geometry change of the downstream. Eventually, the surface energy of a droplet will be surmounted by the combination forces, and the mother droplet will break into two equivalent daughter droplets (Figure 11) [31].

#### Effect of Flow Ratio

**Figure 12:**
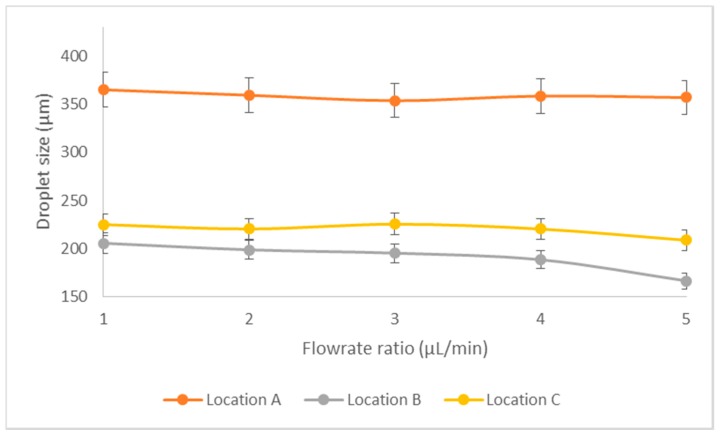
Droplet fission droplet profiles at different flow ratios. *x*-axis denotes the flowrate ratio pair of continuous and dispersed phase flowrate ranging from 2:6 to 6:18 µL/min.

The flow ratio between the continuous and dispersed phase greatly affects the droplet formation as well as fission. With smaller droplets formed at the T-junction at the high velocity, the overall droplet fission size distribution shows an inversely proportional with the increment in flowrates of the dispersed and continuous phases at the constant flow ratio of 1:3. From Figure 12, the droplet fission size shows a direct relationship with respect to the alteration of flowrate ratio. As the flowrate increases, shearing effect is more effective as the continuous phase flow more easily overcomes the surface tension of the disperse phase at the T-junction. Such a phenomenon contributes to more uneven droplet fission distribution as pressure driven mechanism [30] at the splitting channel undergoes a strong competition with the geometry effect [31], which causes the splitting with a bigger droplet size variation.

## 4. Conclusions

In this paper, xurography was introduced as a promising fabrication method that has the capability of rapidly reproducing microfluidic device consisting of both single layer and double layer design with a comparable low installation and fabrication cost in bulk. Bartholomeusz et al. [20] shows that, although its resolution is lower than the standard lithography, the accuracy still falls within 10 µm of the drawn dimensions where the feature variability is less than 20%. However, our work in this paper proved that the error can be reduced to as low as 5% after appropriate optimization of the plotter settings. Utilizing custom-made PDMS aligner in alignment and sealing of two PDMS layers enable the alignment error to be dropped from 2.8% to 1.1%. This error can be further reduced to 0.75% if the glass capillary position control was implemented as we suggested. Experiments carried out using the xurography fabricated devices show convincing results in which all double emulsions produced were well maintained at CV below 10% indicating monodispersity, whereas the droplet fission produced daughter droplets with CV below 5%. We note that the monodispersity of the double emulsions produced is slightly lower as compared to the glass capillary device. Such limitations will be further improved in our future work through the addition of surfactants into the fluid phase. On top of that, flow phenomena studies will be further carried out in depth to better characterize the competition among different forces in droplets fission and break up.

## Figures and Tables

**Table 1:** Relationship between the cutting force and the Y-curvature junction channel width.

Cutting Force	LHS Channel Width (μm) ^1^	Error (%)	RHS Channel Width (μm) ^1^	Error (%)
20	265.01	6.0	177.79	28.9
15	240.97	3.6	201.16	19.5
14	231.10	7.6	184.69	26.1
13	250.60	0.2	202.07	19.1
12	240.52	3.8	218.70	12.5
11	229.59	8.2	198.30	20.7
10	240.15	4.0	200.71	19.7
5	288.45	15.4	154.01	38.4

^1^ Designed channel width for both the right hand side (RHS) and left hand side (LHS) are 250 µm respectively.

**Table 2:** Comparisons of the channel width at each location before and after aligned using aligner and manual alignment.

Position	Default Channel Width (µm)	Channel Width ± Precision (µm, Aligner)	Error Percentage (%, Aligner)	Channel Width (µm, Manual)	Error Percentage (%, Manual)
A	151	151.13 ± 1.13	0.75	151.88 ± 1.13	0.72
B	310	309.60 ± 3.37	1.09	302.12 ± 1.63	2.34
C	310	310.28 ± 3.00	0.97	302.68 ± 1.39	2.83

**Table 3:** Summary of the flowrate ratio and the manipulated parameter.

Group	Flowrate Ratio (μL/min)	Manipulated Flowrate
Group A	1:2:40	Middle phase—continuous cooking oil
	1:4:40	
	1:6:40	
Group B	1:6:40	Outer aqueous phase—continuous yellow dyed water
	1:6:60	

**Table 4:** Coefficient of variance for different types of droplets at each flowrate ratio.

Group	Flowrate Ratio (µL/min)	Inner (Red) Droplets CV (%)	Inner (Blue) Droplets CV (%)	Double Emulsion CV (%)
Group A	1:2:40	17.3	20.1	10.5
	1:4:40	8.0	9.7	5.6
	1:6:40	9.9	13.9	6.7
Group B	1:6:40	9.9	13.9	6.7
	1:6:60	5.9	9.1	9.8

**Table 5:** Success rate of the encapsulation for each combination.

Group	Flowrate Ratio (µL/min)	Percent of 2 Distinct Droplets Encapsulation (1R1B)	Percent of 2 Similar Droplets Encapsulation (2R or 2B)	Percent of Single Droplet Encapsulation (1R or 1B)	% of Zero Encapsulation
Group A	1:2:40	44.2	4.7	51.2	-
	1:4:40	19.1	5.6	69.7	5.6
	1:6:40	35.0	4.0	61.0	-
Group B	1:6:40	35.0	4.0	61.0	-
	1:6:60	49.6	11.9	38.5	0.6
